# Clinicians’ attitude towards a placebo-controlled randomised clinical trial investigating the effect of neuraminidase inhibitors in adults hospitalised with influenza

**DOI:** 10.1186/s12913-018-3122-x

**Published:** 2018-05-02

**Authors:** Naomi Bradbury, Jonathan Nguyen-Van-Tam, Wei Shen Lim

**Affiliations:** 1 Zeeman Institute: Systems Biology and Infectious Disease Epidemiology Research (SBIDER), University of Warwick, Coventry, CV4 7AL UK; 2 School of Life Sciences, University of Warwick, Coventry, CV4 7AL UK; 3 Faculty of Medicine & Health Sciences, University of Nottingham. Clinical Sciences Building, Nottingham City Hospital, Nottingham, NG5 1PB UK; 4 Department of Respiratory Medicine, Nottingham University Hospitals NHS Trust, City Hospital Campus, Nottingham, NG5 1PB UK

**Keywords:** Influenza, Pandemic, Neuraminidase inhibitors, Oseltamivir, Zanamivir, Ethics, Survey, Clinical practice, Equipoise

## Abstract

**Background:**

The value of neuraminidase inhibitors (NAIs) in reducing severe clinical outcomes from influenza is debated. A clinical trial to generate better evidence is desirable. However, it is unknown whether UK clinicians would support a placebo-controlled trial. A survey was conducted to determine the attitude of clinicians towards a clinical trial and their current practice in managing adults admitted to hospital with suspected influenza.

**Methods:**

Senior clinicians (*n* = 50) across the UK actively involved in the care of patients hospitalised with severe respiratory infections and/or respiratory infection research were invited to participate in an on-line survey. Participants were asked their opinion on the evidence for benefit of NAIs in influenza, their current practice in relation to: a) testing for influenza; b) treating empirically with NAIs; and c) when influenza infection is virolologically confirmed, prescribing NAIs.

**Results:**

Thirty-five (70%) of 50 clinicians completed the survey. Respondents were drawn mainly from infectious diseases, intensive care and respiratory medicine. Only 11 (31%) of 35 respondents agreed that NAIs are effective at reducing influenza mortality; 14 (40%) disagreed, 10 (28.6%) neither agreed nor disagreed. When managing adults admitted to non-ICU wards with a respiratory infection during an influenza season, 15 (51.7%) clinicians indicated they would usually perform a test for influenza in greater than 60% of patients but only 9 (31%) would treat empirically with NAIs in greater than 60% of patients. Few clinicians would either test or empirically treat patients presenting with other (non-respiratory infection related) diagnoses. If influenza infection is confirmed, 17 (64.5%) clinicians would prescribe NAIs in greater than 80% of patients with a respiratory infection treated on non-ICU wards Thirty-one (89%) clinicians agreed that a placebo-controlled clinical trial should be conducted and 29 (85%) would participate in such a trial.

**Conclusions:**

There is strong support from UK clinicians for a placebo-controlled trial of NAI treatment in adults hospitalised with suspected influenza. Current variation in medical opinion and clinical practice demonstrates collective equipoise, supporting ethical justification for a trial. Low use of NAIs in the UK suggests randomisation of treatment would not substantially divert patients towards placebo.

**Electronic supplementary material:**

The online version of this article (10.1186/s12913-018-3122-x) contains supplementary material, which is available to authorized users.

## Background

Influenza viruses circulate constantly across the globe, typically causing annual epidemics in temperate regions during the winter months. [1] Annual influenza vaccination is available, which provides some protection against the virus. For the majority of individuals who contract influenza, symptoms are short lived and self-limiting. However, influenza illness can also be severe, particularly among certain susceptible groups such as adults over the age of 65 and individuals with comorbidities. [2] Individuals in these groups are more likely to require hospital admission due to influenza infection and influenza complications may lead to death. [3, 4] In the UK, seasonal influenza is associated with an average of approximately 8000 attributable deaths per year. [5]

Neuraminidase inhibitors (NAIs) such as oseltamivir and zanamivir are licenced and available for the treatment of influenza infection. Based on evidence generated from randomised controlled trials (RCTs) and subsequent meta-analyses, there is general consensus that NAI treatment in uncomplicated seasonal influenza is associated with a reduction in symptom duration of influenza illness by several hours. [6, 7] However, interpretation of the mechanism of this effect differs; the Cochrane group suggested that oseltamivir had no specific antiviral effect, while the Roche-funded review group found that benefit with oseltamivir was limited to the intention-to-treat-infected (influenza confirmed) group suggesting a specific effect on the influenza virus. [6, 7]

Debate regarding the effectiveness of NAIs in reducing severe outcomes amongst patients hospitalised with influenza is even more intense, reflecting the incomplete evidence base in relation to complicated, or severe, influenza. No relevant placebo-controlled RCTs are available to directly inform the debate, instead, the strongest evidence is derived from large observational cohorts, dominated by studies of the use of NAIs for the treatment of pandemic influenza A(H1N1)pdm09. [6–8] An individual participant data (IPD) meta-analysis of data from 78 different observational studies, which included > 29,000 patients, found that the odds of death for adults treated with NAIs within 48 h of symptoms onset, compared to untreated adults was 0.50 (95% CI 0.37–0.67). [9] Although a range of biases were adjusted for within the meta-analysis, further biases may have remained unaccounted for. In reviewing the evidence for NAI use in influenza, an independent steering group of the Academy of Medical Sciences, supported by the Wellcome Trust, acknowledged that although observational studies are generally at higher risk of bias than RCTs, that did not necessarily invalidate the evidence generated by such studies. [10] The report supports the use of NAIs for the treatment of patients hospitalised with influenza, whilst at the same time recognising that the strength of evidence surrounding the effectiveness of NAIs in reducing influenza mortality is sub-optimal and recommending that, in order to improve the evidence base, a RCT investigating the effectiveness of NAI treatment in reducing influenza mortality should be a research priority.

In the UK, NICE Guideline TA 168 (2009) recommends the use of NAIs in ‘at-risk’ persons who present with an influenza-like illness (when influenza is circulating in the community) and who can start treatment within 48 h of the onset of symptoms (or within 36 h for zanamivir treatment in children). [11] Public Health England guidance goes further and recommends that all patients hospitalised with influenza should receive treatment with NAIs; treatment should be started as early as possible without waiting for laboratory confirmation of influenza virus infection. [12] Notably within this recommendation, timing from symptom onset to initiation of NAI is not limited to within 48 h of symptom onset.

Given these, and similar guidelines in other countries, there is a view that a RCT involving patients hospitalised with influenza “*could not ethically evaluate active treatment versus placebo treatment*”. [8] However, in UK clinical practice, adherence to these guidelines is low and NAIs are used infrequently, both in primary care and in hospital settings. This mismatch between clinical practice and guideline recommendation has important implications for clinical care and the feasibility of the conduct of a definitive RCT of NAI treatment in hospitalised patients.

We conducted a nation-wide survey of senior hospital clinicians in the fields of respiratory medicine, intensive care medicine and infectious diseases, who are active in research and clinical care of patients with influenza, to gain some insight into their current practice for managing patients with suspected and confirmed influenza, and their opinion on the necessity for a RCT.

## Methods

An online survey of United Kingdom (UK) clinicians was conducted from 23 February 2017 to 15 March 2017. Clinicians actively involved in the care of patients hospitalised with severe respiratory infections and/or respiratory infection research were approached, including investigators (*n* = 36) involved in the National Institute for Health Research (NIHR) funded pandemic influenza Adjuvant Steroids in Adults with Pandemic Influenza (ASAP) trial. [13] Clinicians not involved in the ASAP trial were selected based on their recognition as opinion-leaders in relation to the management of respiratory tract infections, nationally or within their institutions; all clinicians approached were of Consultant grade.

Fifty clinicians were approached via email containing information about the proposed trial and asking them to complete the survey. The email contained a direct link to the survey which consisted of six multiple-choice questions asking about their current belief in the strength of the evidence surrounding NAI efficacy, their current practice in testing and treating patients hospitalised with suspected or confirmed influenza and if they think that there is the need for a RCT to be conducted (see Additional file 1 Appendix A for survey questions). A follow up email containing the survey information was sent to those who had not responded after the first week.

### Statistical analyses

Results were analysed using R. [14] Descriptive statistics were used to analyse the proportions of respondents and Fisher’s exact test was used to calculate *p* values. A p value of < 0.05 was considered statistically significant.

## Results

### Perceived evidence and need for a trial

There was a good response rate with 35 (70%) of 50 clinicians completing the survey. Intensive Care Medicine specialists and Respiratory Medicine specialists each comprised approximately one third of the respondents (Table 1).

**Table 1:** Survey respondents’ medical specialties (*n* = 35)

Specialty	*n* (%)
Intensive Care Medicine	12 (34.3)
Respiratory Medicine	11 (31.4)
Infectious Diseases	6 (17.1)
Acute Medicine	3 (8.6)
Emergency Medicine	3 (8.6)

**Fig. 1:**
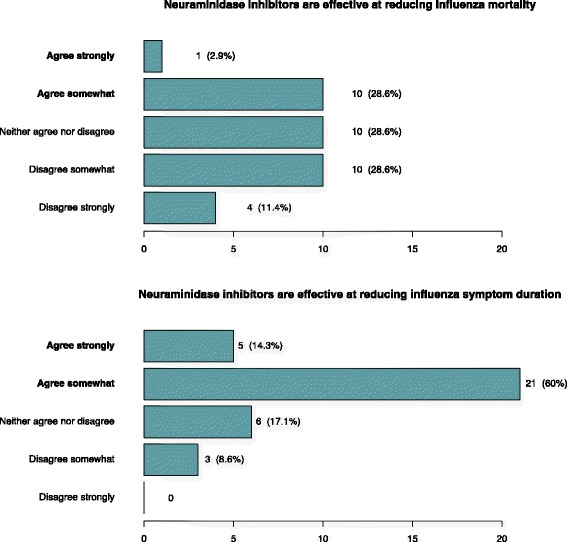
How strongly do you agree or disagree with the following statements about the current evidence for the use of neuraminidase inhibitors (e.g. oseltamivir, zanamivir) in adults hospitalised with suspected influenza?

When asked about their stance regarding the current evidence surrounding NAI effectiveness, 26 (74%) agreed that NAIs were effective at reducing influenza symptom duration, while 6 (17%) neither agreed nor disagreed. But only 11 (31%) agreed that NAIs were effective at reducing influenza mortality while 14 (40%) disagreed, feeling that NAIs are not effective (or unproven) at reducing influenza mortality (Fig. 1).

Thirty-one (89%) clinicians agreed that ‘*a randomised placebo-controlled trial to determine the clinical benefit and cost-effectiveness of neuraminidase inhibitors in adults admitted to hospital with suspected influenza infection should be conducted in the UK’* and 29 (85%) clinicians indicated interest in participating in such a trial.

### Testing for influenza in adults hospitalized during the influenza season

**Fig. 2:**
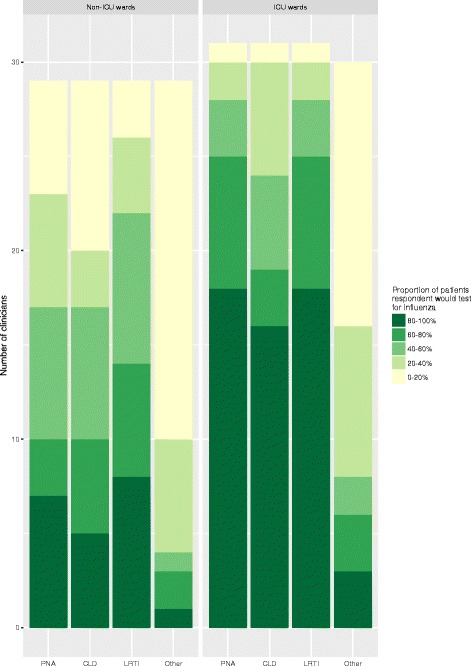
How often do you test for influenza in each of the following groups of adults hospitalised during the influenza season? Legend: PNA – pneumonia, CLD - Exacerbation of chronic lung disease (e.g. COPD, asthma), LRTI – non-pneumonic lower respiratory tract infection, Other – other acute medical illnesses e.g. cardiac failure

Clinicians were asked about their current practice surrounding the testing and treatment of adults admitted to hospital during the influenza season with: a) pneumonia; b) an exacerbation of chronic lung disease; c) non-pneumonic lower respiratory tract infection (LRTI); and d) other diagnoses. For adults admitted to non-ICU wards, 10 (34.5%) clinicians indicated that they would test for influenza in greater than 60% of patients with pneumonia, and 15 (51.7%) clinicians in total would test greater than 60% of patients admitted with any respiratory infection (pneumonia, exacerbation of chronic lung disease or LRTI combined) (Fig. 2). Corresponding figures for adults admitted to ICU were higher; 25 (80.6%) clinicians would test greater than 60% patients with pneumonia (*p* = 0.0003), and 28 (90.3%) clinicians would test greater than 60% of patients admitted with any respiratory infection (*p* = 0.001). Few clinicians would test greater than 60% of adults presenting with other diagnoses, whether admitted to non-ICU wards (*n* = 3 (10.3%) or ICU (*n* = 6 (20.7%)).

### Empirical use of NAIs in adults hospitalized during the influenza season

**Fig. 3:**
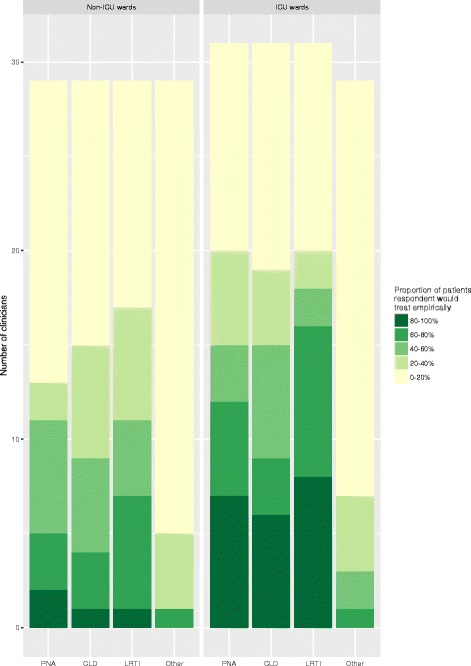
How often do you prescribe neuraminidase inhibitors empirically (i.e. before any influenza test result becomes available) in the following groups of adults hospitalised during the influenza season? Legend: PNA – pneumonia, CLD - Exacerbation of chronic lung disease (e.g. COPD, asthma), LRTI – non-pneumonic lower respiratory tract infection, Other – other acute medical illnesses e.g. cardiac failure

A wide range in the empirical use of NAIs (i.e. when no influenza test result is available) for the treatment of adults admitted with respiratory tract infections was reported. For adults admitted to non-ICU wards, only 5 (17.2%) clinicians would treat empirically with NAIs in greater than 60% of patients with pneumonia and, only 9 (31.0%) clinicians in total would prescribe NAIs empirically to greater than 60% of patients admitted with any respiratory infection. Corresponding figures were higher for adults admitted to ICU; 12 (38.7%) clinicians would treat empirically with NAIs in greater than 60% of patients with pneumonia (*p* = 0.09) and 16 (51.6%) clinicians would treat empirically with NAIs in greater than 60% of patients with any respiratory tract infection (*p* = 0.12) (Fig. 3).

### Use of NAIs when influenza infection is confirmed

**Fig. 4:**
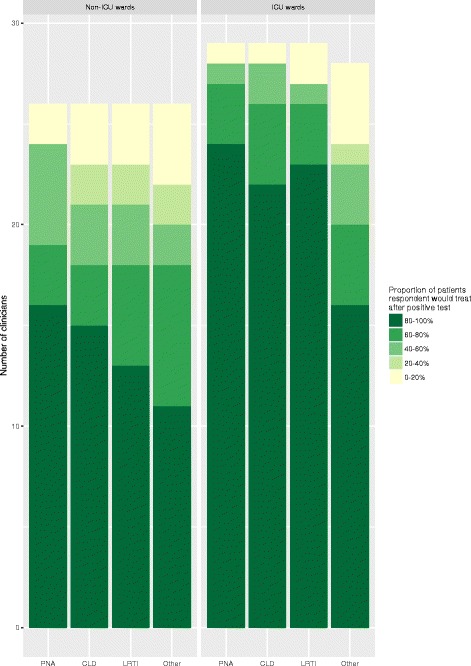
How often do you prescribe neuraminidase inhibitors in each of the following groups of hospitalised adults when influenza infection is confirmed (i.e. influenza test result is positive)? Legend: PNA – pneumonia, CLD - Exacerbation of chronic lung disease (e.g. COPD, asthma), LRTI – non-pneumonic lower respiratory tract infection, Other – other acute medical illnesses e.g. cardiac failure

Most, but not all, clinicians reported that they would prescribe NAIs to greater than 80% of hospitalised adults when influenza infection is confirmed by an influenza test (Fig. 4). Specifically, for adults admitted to non-ICU wards, 16 (61.5%) clinicians would prescribe NAIs in greater than 80% of patients with pneumonia, and 17 (65.4%) clinicians in total would prescribe NAIs in greater than 80% of patients admitted with any respiratory infection.

With regard to adults presenting with illnesses other than a respiratory tract infection in whom influenza infection is confirmed, 11 (42.3%) clinicians would prescribe NAIs in greater than 80% of such patients admitted to non-ICU wards compared to 16 (57.1%) clinicians for such patients admitted to ICU.

## Discussion

The key finding of this survey was the wide range of opinions held by clinicians regarding the effectiveness of NAIs in reducing mortality in patients with influenza; a third of clinicians agreed that NAIs are effective at reducing influenza mortality, a third disagreed and a third neither agreed nor disagreed. This finding carried through to reported clinical practice, with significant variation amongst UK clinicians in relation to the use of NAIs for the treatment of adults hospitalised with suspected influenza infection.

Despite existing PHE guidance recommending a ‘treat and test’ approach to patients admitted to hospital with suspected influenza, the majority of clinicians reported that they would not offer empirical NAI treatment, nor test for influenza infection in most patients regardless of presenting symptoms. Indeed, in patients presenting with pneumonia, in whom it might be expected that empirical NAI treatment would be highest, only 10% of clinicians would offer empirical NAI treatment, and only 35% would test for influenza, in over 60% of ward-based patients. Even when influenza infection is confirmed, only 62% of clinicians would prescribe NAIs in greater than 80% of ward-based patients. A similar trend of very low compliance with PHE guidance was apparent for ward-based patients presenting with less severe respiratory tract infections.

These findings suggest that a placebo-controlled RCT with a 1:1 individual randomisation approach would not lead to an overall diversion of patients from receiving NAI treatment to receiving placebo treatment. Instead, compared with current care, conduct of a RCT would likely lead to more patients with suspected influenza being offered empirical treatment with NAIs and more patients being tested for influenza infection, in accordance with PHE guidance.

For critically ill patients, reported empirical treatment with NAIs was still lower than might be expected with only 38% of clinicians indicating they would empirically treat over 60% of patients presenting with pneumonia. However, the propensity to test for influenza was much higher in critical care; 81% of clinicians would test over 60% of patients with pneumonia.

When confronted by a patient with confirmed influenza infection, the vast majority of clinicians would prescribe NAI treatment for critically ill patients. For ward-based patients, a smaller majority would prescribe NAI treatment; reasons for this difference in opinion were not explored within the survey. Clinicians who are not convinced that NAI treatment provides a survival advantage may feel that confirmation of influenza infection alone does not always justify immediate NAI treatment, but would take into account other factors such time from symptom onset, initial clinical response to supportive treatment, the likelihood of bacterial co-infection, and patient-related prognostic factors. As the odds of death increase, such as in critically ill patients, clinicians may feel more obliged or compelled to offer NAI treatment, despite doubts regarding the beneficial effects of NAIs. Of course, issues regarding whether or not to treat confirmed influenza infection only arise if testing for influenza occurs in the first place.

### Strengths and weaknesses

Although this survey was relatively small, it had a good response rate of 70%, was nation-wide and was representative of a good range of medical specialties. Respondents were senior, experienced clinicians with an interest in influenza and respiratory tract infections who would be expected to be familiar with the existing evidence base. Many respondents were also active researchers in and around the field of influenza. Therefore, the results of the survey represent the views of well-informed and motivated clinicians and clinical academics. We have no reason to expect that clinicians with less interest or expertise in influenza (who are under-represented in this survey) would express markedly different viewpoints.

In a minority of UK hospitals currently, point-of-care influenza testing is available. This may have influenced the responses given by clinicians in relation to both testing for influenza infection and the empirical treatment with NAIs. However, this is likely to be relevant to only a very small proportion of clinicians who responded to the survey and is unlikely to materially alter overall conclusions of the survey. Likewise, some choices in clinical practice, such as testing for influenza, may be influenced by policy within the clinician’s hospital.

### Research implications of findings

From an ethical viewpoint, a clinical trial is generally considered to be justified when there exists ‘an honest, professional disagreement among expert clinicians about the preferred treatment’. [15] If experts are equally divided over the issue, ‘clinical equipoise’ or ‘collective equipoise’ is said to exist and “when deciding if a trial is ethically justified, collective equipoise is considered more important than the individual preferences of attending clinicians”. [16] This survey suggests that in UK clinical practice, notwithstanding existing public health guidance on NAI use, there is indeed clinical and collective equipoise as regards the use of NAIs for adults hospitalised with severe influenza.

In situations where the chances of dying are very high and there is no apparent effective intervention (apart from the trial intervention), it may sometimes be maintained that clinical equipoise alone is not sufficient to justify imposing a randomised clinical trial upon clinical practice; as was argued during the 2014 Ebola outbreak in Africa. [17, 18] Whilst very high mortality rates might pertain in a severe influenza pandemic, in the case of seasonal influenza, mortality rates in hospitalised patients managed according to usual standard of care (SOC) in the UK (which for the most part does not include NAI treatment) ranges between 4 to 23%; similar to mortality rates for community acquired pneumonia. [2, 19] Thus, SOC cannot be considered to be ineffectual in severe seasonal influenza infection, and predictions of high mortality in any individual circumstance are fallible.

The findings from this survey provide a valuable counterpoint to the view that a placebo-controlled RCT of NAI use in adults hospitalised with influenza cannot be conducted on ethical grounds. Instead, there was overwhelming agreement (89%) amongst survey respondents in support of a placebo-controlled RCT trial and given the low current use of NAIs in untested patients, randomisation would not substantially divert patients who would normally be treated with NAIs towards placebo. An adaptive trial design that includes pre-determined interim analyses will enable a large treatment effect (should one exist), such as a 50% reduction in mortality as suggested by the PRIDE IPD meta-analysis, to be detected with good confidence at an earlier stage in the trial. [9] This would mitigate against concerns about unnecessarily exposing large numbers of patients to inferior treatment options. [20] In addition, an adaptive trial design would allow emerging antiviral treatments, including non-NAI antiviral agents, or adjuvant therapies to be tested in a robust and systematic manner. [13, 21]

The downgrading of oseltamivir from the World Health Organization (WHO) list of essential medicines from ‘core’ to ‘complementary’ in September 2017 has fuelled further debate regarding the value of NAI treatment in severe influenza. [22] Such debate is important in raising public awareness regarding the uncertainties in the evidence base as it currently stands. As public perception changes, patient expectations are likely to alter as well. In addition to the clinician survey, we also organised two Patient and Public Involvement (PPI) meetings to discuss the acceptability of a RCT of NAI treatment in hospitalised patients with influenza. At these meetings, PPI members placed a high value on the results from the survey in informing their own views.

Further work is warranted to broaden the consultation to include clinicians based outside of the UK or working within different medical specialties. As this was a self-reported survey, it is possible that clinician responses may differ from their actual practice; measuring actual practice in a hospital setting would improve the strength of these findings.

## Conclusions

UK clinicians strongly support a placebo-controlled RCT of NAI treatment in adults hospitalised with suspected influenza. Current variation in medical opinion and clinical practice is not a sustainable platform for providing patients with suspected influenza the best standard of care possible.

## Additional file

Additional file 1: Appendix – Survey Questions, Survey Questions, Survey questions sent to clinicians. (PDF 56 kb)

## Abbreviations

ICU: intensive care medicine
NAI: neuraminidase inhibitor
NIHR: National Institute for Health Research
PHE: Public Health England
PPI: Patient and Public Involvement
RCT: randomised controlled trial
SOC: standard of care
WHO: World Health Organization
