# Supplementary material for: Clinicians’ attitude towards a placebo-controlled randomised clinical trial investigating the effect of neuraminidase inhibitors in adults hospitalised with influenza
Source: BMC Health Serv Res. 2018 May 2;18:311. doi: 10.1186/s12913-018-3122-x (PMC5930775; doi:10.1186/s12913-018-3122-x)
Supplement: Supplementary file 1 — Appendix – Survey Questions, Survey Questions, Survey questions sent to clinicians. (PDF 56 kb) [file 12913_2018_3122_MOESM1_ESM.pdf]

Influenza trial survey

Page 1

1. How strongly do you agree or disagree with the following statements about the current evidence for the use of neuraminidase inhibitors (e.g. oseltamivir, zanamivir) in **adults hospitalised with suspected influenza**?

	Agree strongly	Agree somewhat	Neither agree nor disagree	Disagree somewhat	Disagree strongly
Neuraminidase inhibitors are effective at reducing influenza mortality	☐	☐	☐	☐	☐
Neuraminidase inhibitors are effective at reducing influenza symptom duration	☐	☐	☐	☐	☐
Neuraminidase inhibitor prescription is worthwhile, even if they are not very effective	☐	☐	☐	☐	☐

Page 2

How often do you **test for influenza** in each of the following groups of adults **hospitalised during the influenza season**?

- Please respond to all questions according to **your current practice**
- Estimate the percentage of patients you would test or treat
- Select N/A if you do not manage such patients, or the question does not apply to you

2. Admitted to non-ICU wards

More info

	0% - 20%	20% - 40%	40% - 60%	60% - 80%	80% - 100%	N/A
Pneumonia	☐	☐	☐	☐	☐	☐
Exacerbation of chronic lung disease (e.g. COPD, asthma)	☐	☐	☐	☐	☐	☐
Non-pneumonic LRTI*	☐	☐	☐	☐	☐	☐
Other acute medical illnesses e.g. cardiac failure	☐	☐	☐	☐	☐	☐

3. Admitted to ICU

	0% - 20%	20% - 40%	40% - 60%	60% - 80%	80% - 100%	N/A
Pneumonia	☐	☐	☐	☐	☐	☐

Exacerbation of chronic lung disease (e.g. COPD, asthma)	☐	☐	☐	☐	☐	☐
Non-pneumonic LRTI	☐	☐	☐	☐	☐	☐
Other acute medical illnesses e.g. cardiac failure	☐	☐	☐	☐	☐	☐

Page 3

How often do you **prescribe** neuraminidase inhibitors **empirically** (i.e. before any influenza test result becomes available) in the following groups of adults **hospitalised during the influenza season**?

4. Admitted to non-ICU wards

	0% - 20%	20% - 40%	40% - 60%	60% - 80%	80% - 100%	N/A
Pneumonia	☐	☐	☐	☐	☐	☐
Exacerbation of chronic lung disease (e.g. COPD, asthma)	☐	☐	☐	☐	☐	☐
Non-pneumonic LRTI	☐	☐	☐	☐	☐	☐
Other acute medical illnesses e.g. cardiac failure	☐	☐	☐	☐	☐	☐

5. Admitted to ICU

	0% - 20%	20% - 40%	40% - 60%	60% - 80%	80% - 100%	N/A
Pneumonia	☐	☐	☐	☐	☐	☐
Exacerbation of chronic lung disease (e.g. COPD, asthma)	☐	☐	☐	☐	☐	☐
Non-pneumonic LRTI	☐	☐	☐	☐	☐	☐

Other acute medical illnesses e.g. cardiac failure	☐	☐	☐	☐	☐	☐

Page 4

How often do you **prescribe** neuraminidase inhibitors in each of the following groups of hospitalised adults when influenza infection is confirmed (i.e. influenza test result is positive)?

6. Admitted to non-ICU wards

	0% - 20%	20% - 40%	40% - 60%	60% - 80%	80% - 100%	N/A
Pneumonia	☐	☐	☐	☐	☐	☐
Exacerbation of chronic lung disease (e.g. COPD, asthma)	☐	☐	☐	☐	☐	☐
Non-pneumonic LRTI	☐	☐	☐	☐	☐	☐
Other acute medical illnesses e.g. cardiac failure	☐	☐	☐	☐	☐	☐

7. Admitted to ICU

	0% - 20%	20% - 40%	40% - 60%	60% - 80%	80% - 100%	N/A
Pneumonia	☐	☐	☐	☐	☐	☐
Exacerbation of chronic lung disease (e.g. COPD, asthma)	☐	☐	☐	☐	☐	☐
Non-pneumonic LRTI	☐	☐	☐	☐	☐	☐

Other acute medical illnesses e.g. cardiac failure	☐	☐	☐	☐	☐	☐

Page 5

8. Do you feel a randomised placebo-controlled trial to determine the clinical benefit and cost-effectiveness of neuraminidase inhibitors in adults admitted to hospital with suspected influenza infection should be conducted in the UK?

- Yes
- No
- Don't know

Page 6

9. Would you be interested in participating in a clinical trial of antiviral use in hospitalised patients?

- ○ Yes
- ○ No
- ○ Maybe

Page 7

10. Please indicate your main specialty

- ○ Infectious diseases
- ○ Medical microbiology
- ○ Emergency medicine
- ○ Intensive care medicine
- ○ Acute medicine
- ○ Respiratory medicine
- ○ Other

10.a. If you selected Other, please specify:

11. If you have any additional comments or questions, please write them in the box below.

12. If you would like to be included in a prize draw for one of 4 x £25 Amazon vouchers, please provide your email address here. We will also inform you of the results of the survey via this address.

Page 8

Thank you for taking the time to complete this survey.
